# Dual FGFR and VEGFR inhibition synergistically restrain hexokinase 2-dependent lymphangiogenesis and immune escape in intrahepatic cholangiocarcinoma

**DOI:** 10.1007/s00535-023-02012-8

**Published:** 2023-07-11

**Authors:** Min Peng, Hui Li, Huan Cao, Yamei Huang, Weiping Yu, Chuanlai Shen, Jinyang Gu

**Affiliations:** 1 Department of Microbiology and Immunology, Medical School of Southeast University, Nanjing, 210009 Jiangsu China; 2 Center for Liver Transplantation, Union Hospital, Tongji Medical College, Huazhong University of Science and Technology, Wuhan, 430022 China; 3 Liver Cancer Institute, Zhongshan Hospital, Fudan University and Key Laboratory of Carcinogenesis and Cancer Invasion, Ministry of Education, Shanghai, 200032 China; 4 Department of Pathology and Pathophysiology, Medical School of Southeast University, Nanjing, 210009 Jiangsu China; 5 Department of Transplantation, Xinhua Hospital Affiliated to Shanghai Jiao Tong University School of Medicine, Shanghai, 200092 China

**Keywords:** Intrahepatic cholangiocarcinoma, Lymphangiogenesis, FGFR, VEGFR3, PD-L1

## Abstract

**Background:**

Therapies for cholangiocarcinoma are largely limited and ineffective. Herein, we examined the role of the FGF and VEGF pathways in regulating lymphangiogenesis and PD-L1 expression in intrahepatic cholangiocarcinoma (iCCA).

**Methods:**

The lymphangiogenic functions of FGF and VEGF were evaluated in lymphatic endothelial cells (LECs) and iCCA xenograft mouse models. The relationship between VEGF and hexokinase 2 (HK2) was validated in LECs by western blot, immunofluorescence, ChIP and luciferase reporter assays. The efficacy of the combination therapy was assessed in LECs and xenograft models. Microarray analysis was used to evaluate the pathological relationships of FGFR1 and VEGFR3 with HK2 in human lymphatic vessels.

**Results:**

FGF promoted lymphangiogenesis through c-MYC-dependent modulation of HK2 expression. VEGFC also upregulated HK2 expression. Mechanistically, VEGFC phosphorylated components of the PI3K/Akt/mTOR axis to upregulate HIF-1α expression at the translational level, and HIF-1α then bound to the HK2 promoter region to activate its transcription. More importantly, dual FGFR and VEGFR inhibition with infigratinib and SAR131675 almost completely inhibited lymphangiogenesis, and significantly suppressed iCCA tumor growth and progression by reducing PD-L1 expression in LECs.

**Conclusions:**

Dual FGFR and VEGFR inhibition inhibits lymphangiogenesis through suppression of c-MYC-dependent and HIF-1α-mediated HK2 expression, respectively. HK2 downregulation decreased glycolytic activity and further attenuated PD-L1 expression. Our findings suggest that dual FGFR and VEGFR blockade is an effective novel combination strategy to inhibit lymphangiogenesis and improve immunocompetence in iCCA.

**Supplementary Information:**

The online version contains supplementary material available at 10.1007/s00535-023-02012-8.

## Introduction

The most common primary liver malignancy after hepatocellular carcinoma, intrahepatic cholangiocarcinoma (iCCA) is a highly lethal neoplasm with a dismal prognosis [1–3]. The only potentially curative option for iCCA is liver resection. However, less than one third of iCCA patients are eligible for curative surgery at the time of diagnosis due to the proclivity iCCA for early lymph node metastasis, and the 5-year survival rate after surgery is only 20–30% because of the high rate of recurrence [4]. Although little is known about the mechanisms promoting iCCA invasiveness, the lymphatic vessels developed during tumour progression provide an important initial route of metastatic dissemination. Much evidence also indicates that tumour-associated lymphangiogenesis is critical for the progression of iCCA [4–6]. Therefore, a deeper understanding of the mechanism underlying lymphangiogenesis might help to identify potential therapeutic targets to prevent iCCA progression and metastasis.

The continuing emergence of immunotherapy is rapidly changing the treatment paradigm for iCCA; however, on account of impaired immunosurveillance, the success rate of iCCA treatment remains disappointing [7]. The lymphatic endothelial cells (LECs) that line all lymphatic vessels, have recently emerged as a component of adaptive immunity, in particular of modulation dendritic cells function and maintaining T cells homeostasis [8]. Under steady-state conditions, LECs express high levels of T-cell inhibitory molecules, including PD-L1 (CD274), which induces apoptosis in tumour-specific CD8^+^ T cells [9]. Many studies have revealed that lactic acid, the final downstream metabolite of anaerobic glycolysis, is responsible for driving PD-L1 expression [10, 11]. Strikingly, lymphangiogenesis involving robust sprouting, migration and proliferation of LECs relies heavily on anaerobic glycolysis for ATP production [12]. Glycolysis is tightly controlled by several rate-limiting enzymes, and hexokinase 2 (HK2) is responsible for the first step in glucose metabolism by catalysing the conversion of glucose to glucose-6-phosphate [13]. Accordingly, HK2-driven glycolysis and the glycolytic end product lactic acid may play pivotal roles in regulating lymphangiogenesis and PD-L1 expression.

The fibroblast growth factor receptors (FGFRs), namely, FGFR1-4, are a family of receptor tyrosine kinases (RTKs) that play important roles in embryonic development, tissue repair, metabolic homeostasis, tumour angiogenesis and proliferation [14]. Recent studies showed that the FGFR signalling pathway regulates lymphangiogenesis by controlling the glycolytic metabolism of LECs [15, 16]. FGFR2 genetic aberrations have been identified in nearly 20% of patients with iCCA. Three selective inhibitors of FGFRs, pemigatinib, infigratinib and futibatinib, have been approved by the US Food and Drug Administration (FDA) for the treatment of locally advanced and metastatic cholangiocarcinoma patients with FGFR2 gene fusion or rearrangement [17–19]. However, some patients with iCCA did not achieve ideal progression-free survival in clinical trials due to the emergence of rapidly acquired resistance [20]. Therefore, a better understanding of the FGFR signalling pathway might help to improve the therapeutic effect of FGFR inhibitors.

Vascular endothelial growth factor (VEGF) has been proven to be overexpressed in biliary tract cancer and has been suggested to be a potential prognostic marker and therapeutic target [21]. SAR131675, a highly selective receptor tyrosine kinase inhibitor of VEGFR3, displays significant antitumour and antimetastatic activities in vivo through inhibition of lymphangiogenesis and tumour-associated macrophage invasion in biliary tract cancer [22]. Although VEGF-C expression serves as an independent and important prognostic factor in iCCA patients and VEGF signalling has been demonstrated to be critical in lymphangiogenesis via Erk1/2 pathways, blocking VEGF does not completely inhibit lymphatic metastasis [23–25]. Therefore, in-depth study of VEGF signalling may identify a new strategy to block lymphatic metastasis of iCCA.

In this study, we found that in LECs and xenograft mice, treatment with either infigratinib or SAR131675 alone only partially inhibited lymphangiogenesis; however, the combination of infigratinib and SAR1331675 almost completely inhibited lymphangiogenesis. Mechanistically, infigratinib inhibits lymphangiogenesis by suppressing HK2 expression regulated by the FGF-MYC-HK2 axis, while SAR131675 inhibits lymphangiogenesis by suppressing HIF-1α-mediated HK2 expression activated by the VEGFC-VEGFR3 signalling system. More importantly, combination therapy significantly suppressed iCCA tumour growth and progression by reducing PD-L1 expression in LECs. In summary, we provide a unique therapeutic approach combining infigratinib and SAR131675 to prevent tumour associated lymphangiogenesis and improve immune activity in iCCA.

## Materials and methods

### Statistical analysis

Statistical analyses were performed using GraphPad Prism 7 software by Student's t test when necessary, and data are expressed as the means ± SDs unless otherwise stated. Overall survival was analysed by the Kaplan–Meier method and the log-rank tests, and correlation analysis was performed using the χ^2^ tests. P values of < 0.05 were considered to indicate significant differences.

Other detailed experimental procedures are described in the Supporting Materials and Methods.

## Results

### The FGF signalling pathway partially regulates lymphangiogenesis by controlling HK2 expression in LECs

**Fig. 1:**
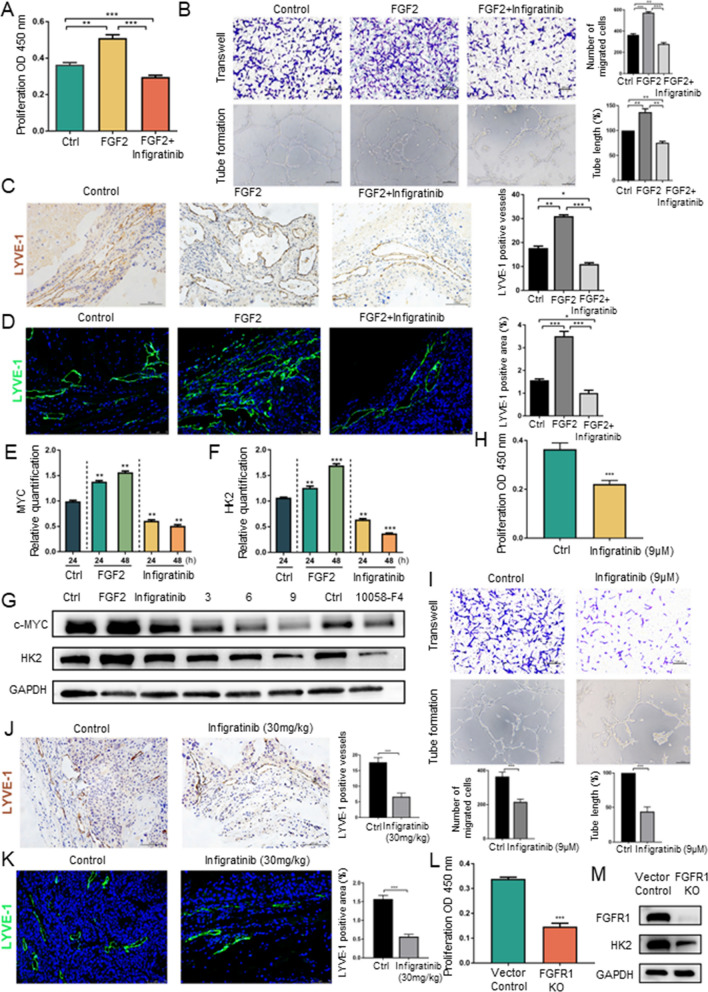
The FGF signalling pathway partially regulates lymphangiogenesis by controlling HK2 expression in LECs. **A** The CCK-8 assay was applied to evaluate the influence of FGF2 and infigratinib on LECs proliferation. **P < 0.01; ***P < 0.001. **B** Transwell and tube formation assays were applied to evaluate the influence of FGF2 and infigratinib on the migration and tubulate activities of LECs. Scale bar: 100 µm. **P < 0.01; ***P < 0.001. **C** Immunohistochemistry analysis of the effect of FGF2 and infigratinib on lymphangiogenesis in iCCA xenograft mice. Scale bar: 100 µm. *P < 0.05; **P < 0.01; ***P < 0.001. **D** Immunofluorescence assays of the effect of FGF2 and infigratinib on lymphangiogenesis in iCCA xenograft mice. Scale bar: 75 µm. *P < 0.05; **P < 0.01; ***P < 0.001. **E** QRT-PCR of LECs was performed to determine the mRNA expression levels of c-MYC after FGF2 or infigratinib treated. **P < 0.01; ***P < 0.001. **F** QRT-PCR of LECs was performed to determine the mRNA expression levels of HK2 after FGF2 or infigratinib treated. **P < 0.01; ***P < 0.001. **G** Western blot analysis of the effect on c-MYC and HK2 expressions after LECs treated with FGF2 or infigratinib or 10058-F4. **H** The CCK-8 assay was applied to evaluate the influence of infigratinib at the concentration of 9 μM on LECs proliferation. ***P < 0.001. **I** Transwell and tube formation assays were applied to evaluate the influence of infigratinib at the concentration of 9 μM on the migration and tubulate activities of LECs. Scale bar: 100 µm. ***P < 0.001. **J** Immunohistochemistry analysis of the effect of infigratinib (30 mg/kg) on lymphangiogenesis in iCCA xenograft mice. Scale bar: 100 µm. ***P < 0.001. **K** Immunofluorescence assays of the effect of infigratinib (30 mg/kg) on lymphangiogenesis in iCCA xenograft mice. Scale bar: 75 µm. ***P < 0.001. **L** The CCK-8 assay was applied to evaluate the proliferation after using CRISPR Cas9 to knockout FGFR1 in LECs. ***P < 0.001. **M** Western blot analysis validated the efficiencies of FGFR1 knockout and HK2 expression after FGFR1 knockout in LECs. Data are shown as the mean ± standard deviation of three independent experiments

The expression of FGFR1 in primary human LECs was detected by flow cytometry. The results showed that FGFR1 was expressed in more than 90% of LECs (Sup Fig. 1A). We further verified that FGFR1 expression in LECs can be stimulated by its classical ligand FGF2 using real-time quantitative PCR and western blot analysis (Sup Fig. 1B, C). The effect of infigratinib, a selective FGFR1-3 inhibitor, has been studied in different tumour cells. However, infigratinib has rarely been used to treat LECs. The Cell Counting Kit-8 assay showed that the inhibitory effect of infigratinib on LEC viability occurred in a dose-dependent and time-dependent manner (Sup Fig. 1D). In the following experiments, we used the cytokine FGF2 and infigratinib to activate and block FGF signalling pathways, respectively. As expected, FGF2 directly induced LEC proliferation, which could be inhibited by infigratinib (Fig. 1A). FGF2 also significantly promoted LEC migration and tube formation, which were suppressed by infigratinib (Fig. 1B). Interestingly, we noted in the tube formation assays that LECs appeared to be aggregated and shrunken after infigratinib treatment. Although tubular structures were still observed, the shape of the formed tubes was different from that in the control group (Fig. 1B). To further confirm the effect of FGFR signalling on lymphangiogenesis in vivo, we established mouse xenograft models with HuCCT1 iCCA cell lines as described previously. LYVE-1 expression was measured by immunohistochemistry and immunofluorescence to analyse lymphangiogenesis in vivo. The results showed that FGF2 significantly promoted an increase in lymphatic vessels and dilation of the lumen, while infigratinib inhibited lymphangiogenesis in vivo (Fig. 1C, D). A previous report indicated that FGF-dependent regulation of lymphatic development occurs through endothelial metabolism driven by MYC-dependent regulation of HK2 expression [16]. The FGF-MYC-HK2 axis is the crucial driver of glycolytic metabolism in LECs. Our data showed that FGF2 promoted the expression of c-MYC and HK2, while infigratinib greatly inhibited the expression of c-MYC and HK2 at the mRNA level (Fig. 1E, F). Consistent with the qPCR results, western blot analysis confirmed that c-MYC and HK2 expression was increased by FGF2 and inhibited by infigratinib (Fig. 1G). With the increase in the concentration of infigratinib, the expression of c-MYC and HK2 showed a graded downwards trend (Fig. 1G). To further verify the role of c-MYC in the FGF-MYC-HK2 axis, the c-MYC inhibitor 10058-F4 was used to treat LECs [26]. When c-MYC was inhibited, the expression of HK2 was significantly suppressed (Fig. 1G). Given the above findings, we verified that FGF2 and infigratinib can promote and inhibit lymphangiogenesis, respectively, by controlling the FGF-MYC-HK2 axis. We continued to study the inhibitory effects of infigratinib alone on lymphangiogenesis by treatment of LECs with infigratinib at the maximum therapeutic dose (9 μM). We found that when LECs were treated with infigratinib at a concentration of 9 μM, the proliferation, migration and tube formation ability of the LECs could be inhibited but not completely blocked (Fig. 1H, I). Moreover, the immunohistochemistry and immunofluorescence results showed that infigratinib (30 mg/kg) inhibited lymphangiogenesis in the iCCA mouse model but did not completely block lymphangiogenesis (Fig. 1J, K). Reanalysis of the western blot results in Fig. 1G also showed that HK2 expression could not be completely blocked even after treatment with the maximum dose of infigratinib. We further utilized CRISPR-Cas9 gene editing to knock out FGFR1 in LECs and found that LEC growth and HK2 expression were not completely inhibited (Fig. 1L, M), consistent with the above mentioned effects of infigratinib. These above data promoted us to consider whether there are any other molecular pathways that regulate HK2 expression and then affect lymphangiogenesis.

### The VEGF signalling system partially regulates HK2 expression in LECs and then affects lymphangiogenesis

**Fig. 2:**
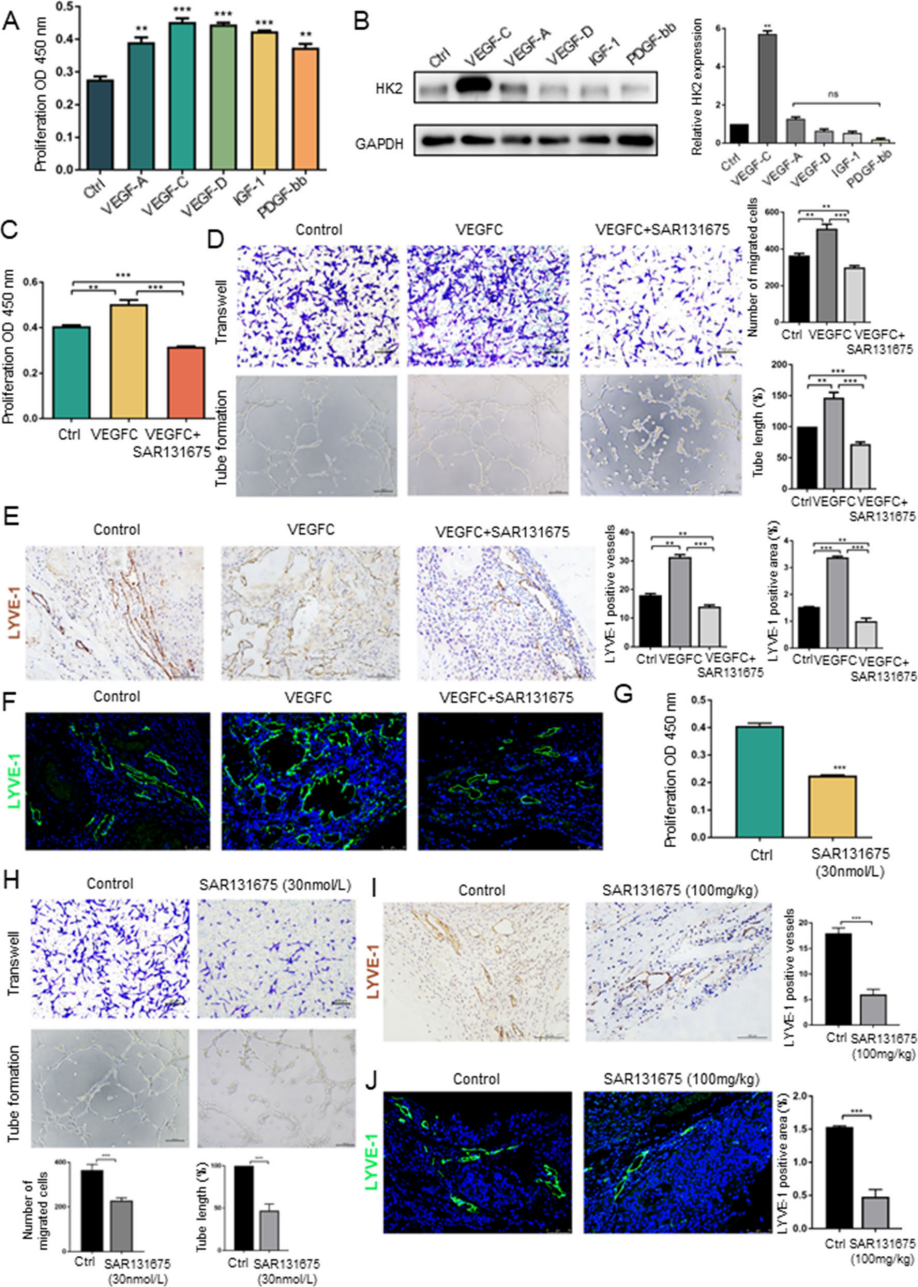
The VEGF signalling system partially regulates HK2 expression in LECs and then affect lymphangiogenesis. **A** The CCK-8 assay was applied to evaluate the influence of VEGF-A, VEGF-C, VEGF-D, IGF-1 and PDGF-bb on LECs proliferation. **P < 0.01; ***P < 0.001. **B** Western blot analysis of the effect on HK2 expression after LECs treated with VEGF-C or VEGF-A or VEGF-D or IGF-1 or PDGF-bb. **P < 0.01; ns, not significant. **C** The CCK-8 assay was applied to evaluate the influence of VEGFC and SAR131675 on LECs proliferation. **P < 0.01; ***P < 0.001. **D** Transwell and tube formation assays were applied to evaluate the influence of VEGFC and SAR131675 on the migration and tubulate activities of LECs. Scale bar: 100 µm. **P < 0.01; ***P < 0.001. **E** Immunohistochemistry analysis of the effect of VEGFC and SAR131675 on lymphangiogenesis in iCCA xenograft mice. Scale bar: 100 µm. **P < 0.01; ***P < 0.001. **F** Immunofluorescence assays of the effect of VEGFC and SAR131675 on lymphangiogenesis in iCCA xenograft mice. Scale bar: 75 µm. **P < 0.01; ***P < 0.001. **G** The CCK-8 assay was applied to evaluate the influence of SAR131675 at the concentration of 30 nM on LECs proliferation. ***P < 0.001. **H** Transwell and tube formation assays were applied to evaluate the influence of SAR131675 at the concentration of 30 nM on the migration and tubulate activities of LECs. Scale bar: 100 µm. ***P < 0.001. **I** Immunohistochemistry analysis of the effect of SAR131675 (100 mg/kg) on lymphangiogenesis in iCCA xenograft mice. Scale bar: 100 µm. ***P < 0.001. **J** Immunofluorescence assays of the effect of SAR131675 (100 mg/kg) on lymphangiogenesis in iCCA xenograft mice. Scale bar: 75 µm. ***P < 0.001. Data are shown as the mean ± standard deviation of three independent experiments

In addition FGF-2, several other growth factors, including vascular endothelial growth factor-A (VEGF-A), vascular endothelial growth factor-C (VEGF-C), vascular endothelial growth factor-D (VEGF-D), insulin-like growth factors-1(IGF-1) and platelet-derived growth factor-bb (PDGF-bb), can induce lymphangiogenesis [27–31]. CCK-8 and western blot assays demonstrated that all the above cytokines can induce LEC proliferation; however, only VEGFC increased HK2 expression (Fig. 2A, B). Next, we investigated how VEGFC affects lymphangiogenesis by controlling HK2 expression. VEGFC is a member of the VEGF family, that can specifically activate VEGFR3 in LECs. The expression of VEGFR3 was detected by flow cytometry, and the results confirmed that VEGFR3 was widely expressed in LECs (Sup Fig. 2A). SAR131675, a highly selective inhibitor of VEGFR3, has a dose and time-dependent effect on the proliferative ability of LECs (Sup Fig. 2B). VEGFC and SAR131675 were used to activate and block the VEGF signalling pathway, respectively, in subsequent assays. We observed that LEC proliferation was significantly enhanced by VEGFC and suppressed by SAR131675 (Fig. 2C). In our migration and tube formation assays, stimulation of LECs with VEGFC led to significantly increased migration and induced denser tube formation, but migration and tube formation were obviously inhibited by SAR131675 (Fig. 2D). To further explore how the VEGF signalling system regulates lymphangiogenesis in vivo, mice in the iCCA model were treated with VEGFC and SAR131675. The immunohistochemical and immunofluorescence staining results further confirmed that VEGFC dramatically promoted lymphangiogenesis (stained by LYVE-1 antibody) whereas SAR131675 efficiently abrogated lymphangiogenesis induced by VEGFC in vivo (Fig. 2E, F). Given that the VEGFC/VEGFR3 axis is a classical signalling pathway regulating lymphangiogenesis, we sought to determine whether inhibition of the VEGF signalling system alone can completely abrogate lymphangiogenesis. We later treated LECs and xenograft mice with SAR131675 at the maximum dose (30 nM in vitro and 100 mg/kg in vivo). Consistent with the effect of infigratinib on LECs, SAR131675 did not completely abolish the proliferation, migration and tube formation of LECs at concentrations up to 30 nM (Fig. 2G,>H), although the inhibitory effect was prominent. The results of immunohistochemical and immunofluorescence staining for LYVE-1 also showed that lymphangiogenesis cannot be completely inhibited by SAR131675 in vivo (Fig. 2I, J). In summary, the VEGF signalling system partially regulates HK2 expression and then affects lymphangiogenesis.

### The VEGFC signalling system regulates HK2 expression by phosphorylating PI3K/Akt/mTOR pathway proteins that regulate HIF-1α expression

**Fig. 3:**
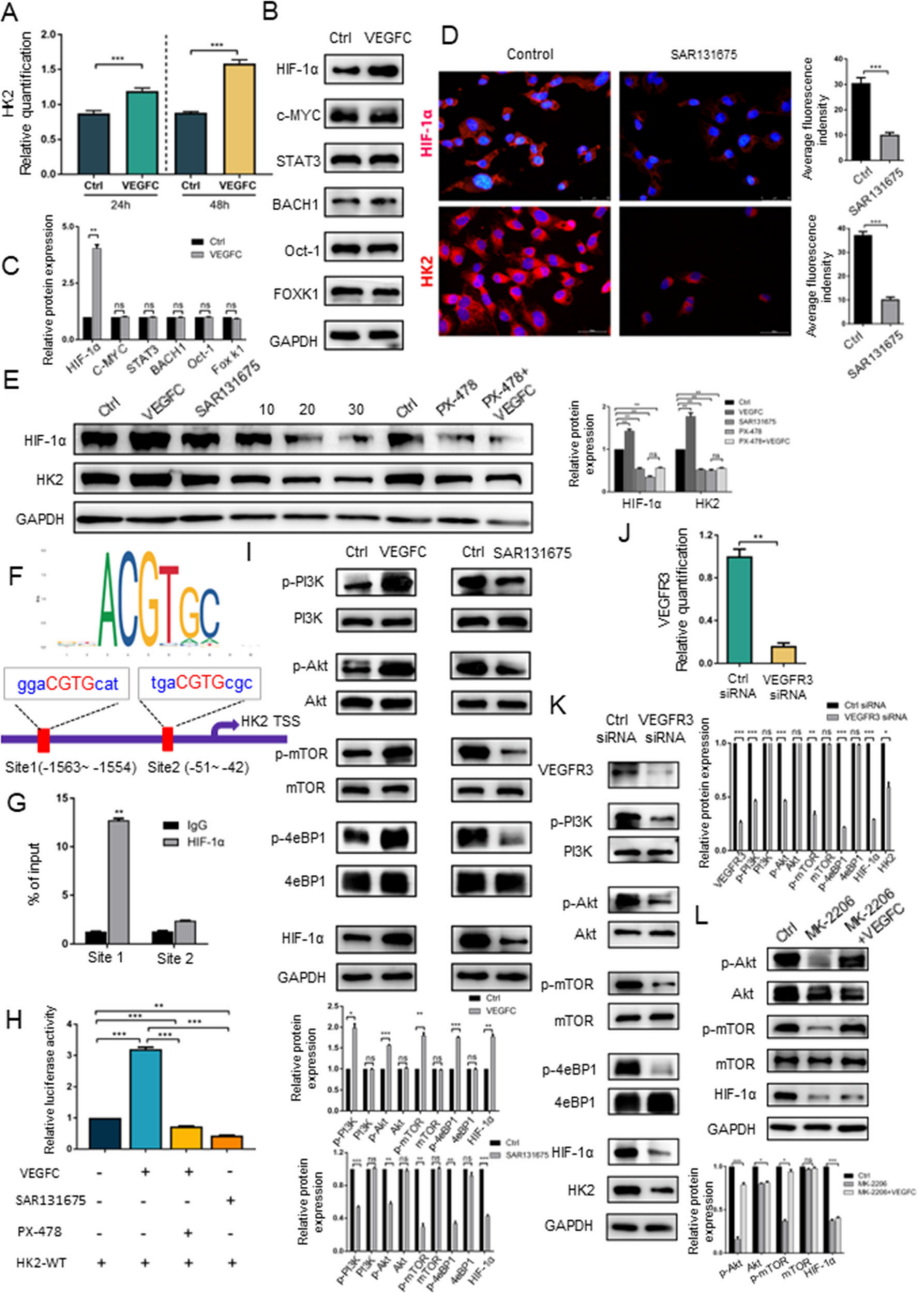
The VEGFC signalling system regulates HK2 expression by phosphorylating PI3K/Akt/mTOR pathway proteins that regulate HIF-1α expression. **A** QRT-PCR of LECs was performed to determine the mRNA expression levels of HK2 after VEGFC treated. **B**, **C** Western blot analysis of the effect of VEGFC stimulation on the expression of transcription factor proteins in LECs. **P < 0.01; ns, not significant. **D** Immunofluorescence assays showing the HIF-1α and HK2 expressions after SAR131675 treated LECs. Scale bar: 50 µm. ***P < 0.001. **E** Western blot analysis of the effect on HIF-1α and HK2 expressions after LECs treated with VEGFC or SAR131675 or PX-478 or PX-478 plus VEGFC. **P < 0.01. **F** The bioinformatics analysis indicated the putative hypoxia response elements (HRE) (CGTG) on the upstream promoter region of HK2 for HIF-1α. **G** Chromatin immunoprecipitation (ChIP) results indicated the binding of HIF-1α on the site 1 of HK2 promoter. **P < 0.01. **H** Luciferase reporter assays were performed to detect the transcriptional activity of HIF-1α on HK2 in LECs after VEGFC and/or PX-478 or SAR131675 treatment. ***P < 0.001. **I** Western blot analysis of the phosphorylation on PI3K/Akt/mTOR axis proteins after treated LECs with VEGFC or SAR131675. *P < 0.05; **P < 0.01; ***P < 0.001; ns, not significant. **J** The efficiency of VEGFR3 knockdown was validated by qRT-PCR in LECs. ***P < 0.001. **K** Knockdown of VEGFR3 in LECs inhibited PI3K/Akt/mTOR axis phosphorylation and HIF-1α and HK2 protein expression levels. *P < 0.05; **P < 0.01; ***P < 0.001; ns, not significant. **L** Western blot analysis of the effect on p-Akt, Akt, p-mTOR, mTOR and HIF-1α expressions after LECs treated with MK-2206 as well as MK-2206 plus VEGFC. *P < 0.05; **P < 0.01; ***P < 0.001; ns, not significant. Data are shown as the mean ± standard deviation of three independent experiments

The qPCR results indicated that VEGFC upregulated HK2 expression at the transcriptional level (Fig. 3A). It has been proven that transcription factors such as HIF-1α, c-MYC, STAT3, BACH1, Oct1 and Foxk1 can activate the transcription of hexokinase II [32–36]; however, we found that among these transcription factors, only HIF-1α can be activated by VEGFC in LECs (Fig. 3B, C). Moreover, fluorescence staining of LECs confirmed that HIF-1α and HK2 expression decreased significantly after SAR131675 treatment (Fig. 3D). Furthermore, western blot analysis showed that the expression of both HIF-1α and HK2 was significantly upregulated and downregulated when LECs were treated with VEGFC and SAR131675, respectively (Fig. 3E). As the treatment concentration of SAR131675 increased, the expression of HIF-1α and HK2 was gradually inhibited (Fig. 3E). To further verify the key role of HIF-1α in the regulation of HK2 expression by the VEGFC signalling system, we used PX-478, a HIF-1α inhibitor, to treat LECs [26]. The western blot results showed that PX-478 treatment reduced the expression of HIF-1α and HK2 and that HK2 expression could not be rescued by VEGFC treatment (Fig. 3E). Another remaining question was how the transcription factor HIF-1α regulates HK2. The bioinformatics analysis indicated that HIF-1α had the putative Hypoxia Response Elements (HRE) (CGTG) on the upstream promoter region of HK2 (Fig. 3F). Chromatin immunoprecipitation (ChIP) results indicated that HIF-1α could bind with the site 1 of HK2 promoter (Fig. 3G). The transcriptional activity of HIF-1α towards HK2 was also quantified in luciferase reporter assays. LECs were cotransfected with the HIF-1α overexpression plasmid and a plasmid containing the wild-type HK2 promoter sequence, a plasmid containing the mutant HK2 promoter sequence or HK2 vector control. We measured luciferase activity and observed a distinct difference in activity in the HIF-1α overexpression and wild-type HK2 promoter plasmid cotransfection group (Sup Fig. 3B). Treatment with VEGFC resulted in an approximately 3.2-fold increase in HK2 reporter gene activity, and this increased was attenuated by treatment with the HIF-1α inhibitor PX-478 (Fig. 3G). Moreover, SAR131675 treatment significantly reduced luciferase reporter activity compared with that in the control group (Fig. 3G). These data suggested that VEGFC induced transcriptional activation of the HK2 gene through the activation of HIF-1α, while SAR131675 inhibited this transcriptional activation. Notably, the HIF-1α binding region is located at least 1 kb from the intronic c-MYC binding region, suggesting that HIF-1α and c-MYC could independently transactivate the HK2 gene [37]. Considering these results collectively, we identified HIF-1α as a key transcription factor in VEGFC-mediated-regulation of HK2 expression. Next, we investigated how VEGFC regulates HIF-1α expression. To characterize the mechanism by which the VEGFC signalling system regulates HIF-1α expression, a real-time quantitative PCR assay was conducted. The qPCR results showed that the HIF-1α mRNA levels in the VEGFC treatment group and SAR131675 treatment group were similar to those in the control group (Sup Fig. 3A), consistent with the previous reports indicating that the PI3K/Akt/mTOR signalling pathway regulates HIF-1α synthesis at the translational level [38, 39]. Western blotting was carried out to examine whether the VEGF pathway regulates the PI3K/Akt/mTOR signalling pathway. The results suggested that VEGFC increased the levels of p-PI3K, p-Akt, p-mTOR and p-4eBP1 without affecting the total protein levels of PI3K, Akt, mTOR and 4eBP1; on the other hand, SAR131675 significantly reduced the phosphorylation of these proteins and had no effect on the total protein levels. Upregulation and downregulation of PI3K-Akt-mTOR signalling further led to induction and suppression of HIF-1α expression, respectively (Fig. 3H). VEGFR3 knockdown with siRNA was carried out to further verify the regulation of HIF-1α expression by the VEGF pathway. We first examined the mRNA level of VEGFR3 in LECs after transfection with VEGFR3 siRNA. The results showed that the level of VEGFR3 was significantly decreased after transfection with the siRNA (Fig. 3I). Next, the western blot analysis results proved that VEGFR3 expression was also notably suppressed at protein level (Fig. 3J). Then, we evaluated the impact of VEGFR3 knockdown on the PI3K/AKT/mTOR pathway. As shown by the results, knockdown of VEGFR3 with siRNA significantly decreased the levels of phosphorylated PI3K, Akt, mTOR and 4eBP1 without affecting the total protein expression levels (Fig. 3J). As expected, knockdown of VEGFR3 with siRNA significantly decreased HIF-1α and HK2 expression (Fig. 3J). Eukaryotic translation initiation factor 4E- (eIF-4E-) binding protein (4eBP1) is a negative regulator of 5' cap-dependent mRNA translation. Phosphorylated 4eBP1 can be dissociated from eukaryotic translation initiation factor 4E (eIF4E) to relieve its inhibition of protein synthesis. Simultaneously, P70 S6 kinase can be phosphorylated and activate 40S ribosomal proteins. Activation of both eIF-4E and the 40S ribosomal protein initiates translation of the HIF-1α mRNA [40, 41]. To further confirm that the PI3K/Akt/mTOR pathway is involved in the synthesis of HIF-1α, MK-2206 was used as an Akt inhibitor to treat LECs. We found that MK-2206 reduced HIF-1α expression by blocking the phosphorylation of Akt pathway proteins, an effect that could not be rescued by VEGFC (Fig. 3K). Collectively, these data indicated that VEGFC regulates HIF-1α-mediated HK2 expression via the PI3K/Akt/mTOR pathway.

### Dual FGFR and VEGFR inhibition almost completely inhibits lymphangiogenesis in vitro and in vivo

**Fig. 4:**
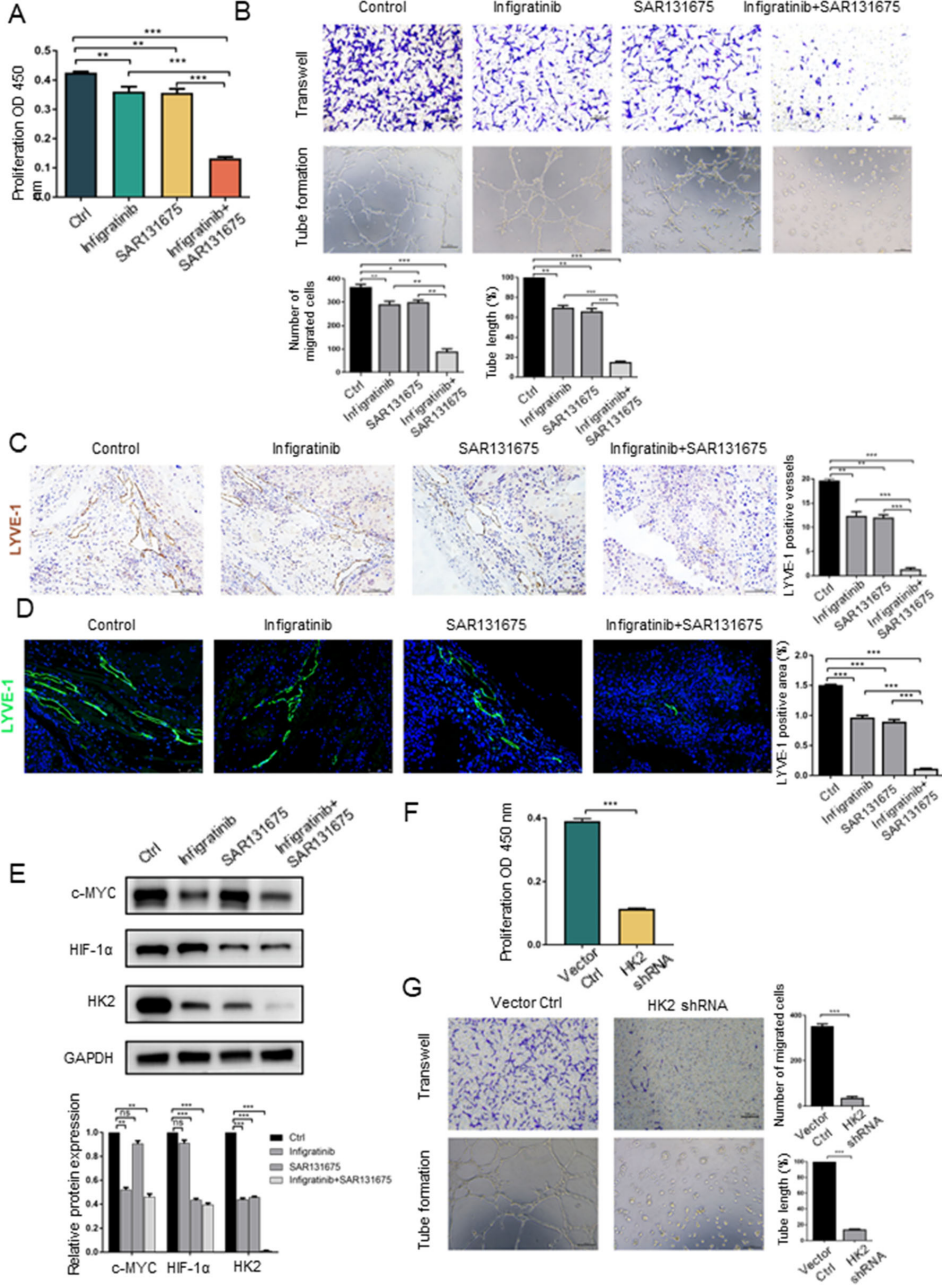
Dual FGFR and VEGFR inhibition almost completely inhibit lymphangiogenesis in vitro and vivo. **A** The CCK-8 assay was applied to evaluate the influence of infigratinib and SAR131675 treated alone or combination on LECs proliferation. **P < 0.01; ***P < 0.001. **B** Transwell and tube formation assays were applied to evaluate the influence of infigratinib and SAR131675 treated alone or combination on the migration and tubulate activities of LECs. Scale bar: 100 µm. *P < 0.05; **P < 0.01; ***P < 0.001. **C** Immunohistochemistry analysis of the effect of infigratinib and SAR131675 treated alone or combination on lymphangiogenesis in iCCA xenograft mice. Scale bar: 100 µm. **P < 0.01; ***P < 0.001. **D** Immunofluorescence assays of the effect of infigratinib and SAR131675 treated alone or combination on lymphangiogenesis in iCCA xenograft mice. Scale bar: 75 µm. ***P < 0.001. **E** Western blot analysis of the effect on c-MYC, HIF-1α and HK2 expressions after infigratinib and SAR131675 treated LECs alone or combination. **P < 0.01; ***P < 0.001; ns, not significant. **F** The CCK-8 assay was applied to evaluate the influence on LECs proliferation after knockdown HK2 with shRNA. ***P < 0.001. **G** Transwell and tube formation assays were applied to evaluate the influence of HK2 knockdown on the migration and tubulate activities of LECs. Scale bar: 100 µm. ***P < 0.001. Data are shown as the mean ± standard deviation of three independent experiments

As mentioned above, infigratinib and SAR131675 partially inhibited lymphangiogenesis by reducing expression of the HK2, a finding that has been studied separately. We found that even the maximum dose of a single drug could not completely inhibit lymphangiogenesis, and the tumour-bearing mice showed emaciation, which led to rapid death under this treatment dose. Therefore, we decided to examine whether infigratinib in combination with SAR131675 would exert the ideal inhibitory effect on lymphangiogenesis in vitro and in vivo. The CCK-8 assay showed that infigratinib and SAR131675 in combination dramatically reduced the proliferative ability of LECs compared to either single drug at a lower treatment dose (infigratinib at 3 μM and SAR131675 at 10 nM) (Fig. 4A). Migration and tube formation assays were subsequently conducted to evaluate the effects of this treatment dose. The results showed that treatment of LECs with infigratinib and SAR131675 in combination significantly decreased their migratory ability compared to either single drug, and that surprisingly only a few cells migrated through the polycarbonate membrane (Fig. 4B). The combination treatment also completely inhibited tube formation of LECs. LECs were scattered throughout the imaging field, without a regular tubular shape (Fig. 4B). Furthermore, immunohistochemical and immunofluorescence staining of LYVE-1 demonstrated that the combination of infigratinib and SAR131675 almost completely inhibited lymphangiogenesis in iCCA-bearing mice compared to either single drug (Fig. 4C, D). The optimal combination treatment dosing regimen in vivo is daily oral treatment with infigratinib (15 mg/kg) and SAR131675 (50 mg/kg), at concentrations far below the maximum therapeutic doses of infigratinib and SAR131675 alone. Moreover, western blot analysis showed that the expression of HK2 was almost completely inhibited when LECs were treated with infigratinib combined with SAR131675 (Fig. 4E). It is worth noting that the expression of c-MYC and HIF-1α did not differ in the combination treatment group compared with the infigratinib and SAR131675 single drug groups, indicating that the FGF and VEGF signalling systems independently regulate HK2 expression through c-MYC and HIF-1α, respectively (Fig. 4E). Taken together, these results indicated that infigratinib in combination with SAR131675 almost completely inhibited lymphangiogenesis at concentrations lower than the corresponding single-agent doses in vitro and in vivo. In addition, we used short hairpin RNAs to decrease the expression of HK2 (Sup Fig. 4A, B) and then showed the critical role of HK2 in regulating lymphangiogenesis by influencing the proliferation, migration and tube formation ability of LECs (Fig. 4F, G). These findings were consistent with our previous findings.

### FGFR1 and VEGFR3 expression is related to hexokinase II expression in hLECs, and are more prone to induce lymphangiogenesis and lymphatic metastasis in iCCA patients

**Fig. 5:**
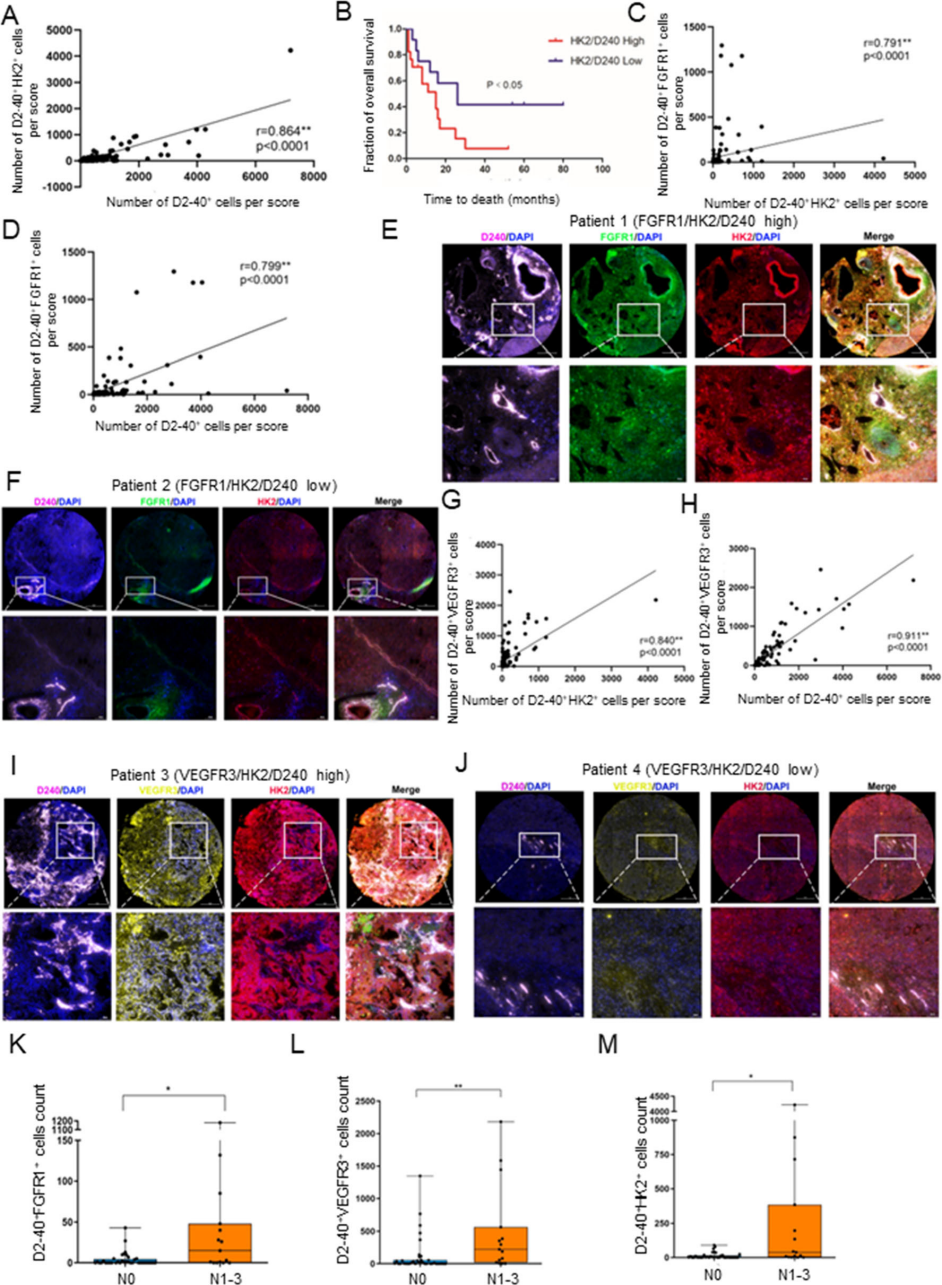
FGFR1 and VEGFR3 expression is related to hexokinase II expression in hLECs, and are more prone to induce lymphangiogenesis and lymphatic metastases in iCCA patients. **A** Pearson’s correlation coefficient quantified the correlation between D2-40^+^HK2^+^ cells and D2-40^+^ cells. **B** Kaplan–Meier analysis of the overall survival of D2-40^+^HK2^+^ iCCA patients. *P* values < 0.05 are considered significant. **C** Pearson’s correlation coefficient quantified the correlation between D2-40^+^FGFR1^+^ cells with D2-40^+^HK2^+^ cells. **D** Pearson’s correlation coefficient quantified the correlation between D2-40^+^FGFR1^+^ cells and D2-40^+^ cells. **E** Representative images of iCCA patient with high expression of FGFR1, HK2 and D2-40 in hLECs. **F** Representative images of iCCA patient with low expression of FGFR1, HK2 and D2-40 in hLECs. **G** Pearson’s correlation coefficient quantified the correlation between D2-40^+^VEGFR3^+^ cells with D2-40^+^HK2^+^ cells. **H** Pearson’s correlation coefficient quantified the correlation between D2-40^+^VEGFR3^+^ cells and D2-40^+^ cells. **I** Representative images of iCCA patient with high expression of VEGFR3, HK2 and D2-40 in hLECs. **J** Representative images of iCCA patient with low expression of VEGFR3, HK2 and D2-40 in hLECs. **K** D2-40^+^FGFR1^+^ cells induce lymph node metastasis. *P < 0.05. **L** D2-40^+^VEGFR3^+^ cells induce lymph node metastasis. **P < 0.01. **M** D2-40^+^HK2^+^ cells induce lymph node metastasis. *P < 0.05. Data are shown as the mean ± standard deviation of three independent experiments

To thoroughly understand tumour-associated lymphangiogenesis in patients with iCCA, multiplex immunohistochemistry (mIHC) staining of a tissue microarray composed of 155 samples of tumour and 5 samples of adjacent tissue from 155 iCCA patients was analysed. D2-40 antibody staining of human lymphatic endothelial cells (hLECs) was used to help locate lymphatic vessels. The numbers of hLECs with FGFR1 (green), VEGFR3 (yellow), HK2 (red) and D2-40 (purple) expression were determined in each sample. Subcellular colocalization analysis of FGFR1, VEGFR3, HK2, and D2-40 was performed to identify the expression of these receptors in lymphatic vessels. The results are presented in Table S1. Pearson correlation analysis quantitatively showed that the number of D2-40^+^HK2^+^ cells was significantly correlated with that of D2-40^+^ cells (P-value < 0.0001) (Fig. 5A, Sup Fig. 5A). Kaplan–Meier survival analysis proved that a high expression of HK2 in lymphatic vessels was significantly associated with worse prognosis (Fig. 5B). High expression of FGFR1 in lymphatic vessels was also significantly associated with worse prognosis (Sup Fig. 5F). We further examined the relationship between FGFR1 and HK2 expression in lymphatic vessels. The results showed that the number of D2-40^+^FGFR1^+^ cells was substantially correlated with that of D2-40^+^HK2^+^ cells (P-value < 0.0001) (Fig. 5C, Sup Fig. 5B). Moreover, the number of D2-40^+^FGFR1^+^ cells was significantly correlated with that of D2-40^+^ cells (P-value < 0.0001) (Fig. 5D, Sup Fig. 5C). Representative samples mIHC staining are shown in Fig. 5 E and F. Kaplan–Meier survival analysis proved that a high expression of VEGFR3 in lymphatic vessels was significantly associated with worse prognosis (Sup Fig. 5G). The correlation between VEGFR3 and HK2 expression in lymphatic vessels was also quantified in the tissue microarray. The analysis indicated that the number of D2-40^+^VEGFR3^+^ cells was strongly correlated with that of D2-40^+^HK2^+^ cells (P-value < 0.0001) (Fig. 5G, Sup Fig. 5D). Moreover, the number of D2-40^+^VEGFR3^+^ cells was also significantly correlated with that of D2-40^+^ cells (P-value < 0.0001) (Fig. 5H, Sup Fig. 5E). Representative samples with mIHC staining are shown in Fig. 5I and J. The above results clarify that FGFR1 and VEGFR3 in lymphatic vessels are positively correlated with lymphangiogenesis and that this process is related to HK2 in human lymphatic endothelial cells. We then analysed the correlations of D2-40^+^ cells with HK2^+^ cells, FGFR1^+^ cells and VEGFR3^+^ cells and found that D2-40^+^ cells were also substantially correlated with all three (Sup Fig. 5H, I, J). We further analysed the relationships between D2-40^+^FGFR1^+^, D2-40^+^VEGFR3^+^, D2-40^+^HK2^+^ cells and TNM stage. The results showed that increased number of D2-40^+^FGFR1^+^, D2-40^+^VEGFR3^+^ and D2-40^+^HK2^+^ cells are associated with increased lymph node metastasis in iCCA (Fig. 5K, L, M, Sup Table S2, S3 S4), which hinted that lymphangiogenesis may correlated with lymph node metastasis in iCCA.

### Dual FGFR and VEGFR inhibition improves antitumour immunity by downregulating PD-L1 expression in LECs

**Fig. 6:**
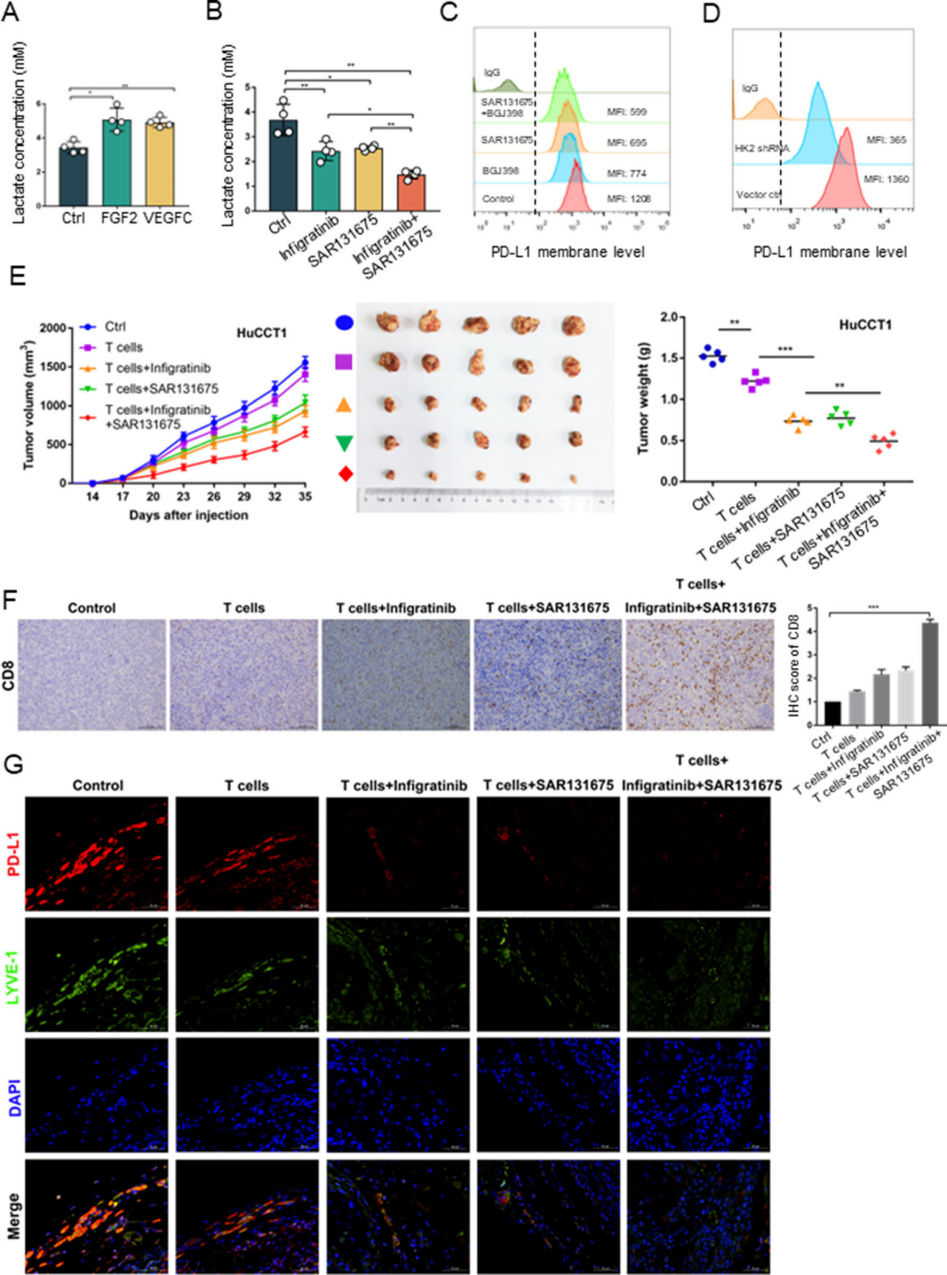
Dual FGFR and VEGFR inhibition improves antitumour immunity by downregulating PD-L1 expression in LECs. **A** Measurement of lactate concentration to assess the influence of FGF2 and VEGFC on lactate production in LECs. *P < 0.05; **P < 0.01. **B** Measurement of lactate concentration to assess the influence of infigratinib or/and SAR131675 on lactate production in LECs. *P < 0.05; **P < 0.01; ***P < 0.001. **C** Flow cytometry was applied to detect the effect of infigratinib and SAR131675 treated alone or combination on membrane PD-L1 expression in LECs. MFI: Mean Fluorescence Indensity. **D** Flow cytometry was applied to detect membrane PD-L1 expression on LECs after transfected with HK2 shRNA and vector plasmid. MFI: Mean Fluorescence Indensity. **E** Mice were injected with human iCCA cells (HuCCT1 cells). The control animals received no further injections. The experimental treatments entailed injections with T cells or T cells in combination with infigratinib or SAR131675 or infigratinib as well as SAR131675 combination treatments. The illustrated data represent tumor volumes and weights (five mice in each group). The day of tumor cell injection was counted as day 0. The tumors were excised and photographed 35 days after injecting the tumor cells. **F** CD8^+^ T-cell infiltration in tumors was measured by immunohistochemistry. Scale bar: 100 μm. ***P < 0.001. **G** PD-L1 and LYVE-1 expression in LECs was measured by immunofluorescence. Scale bar: 50 µm

It has been reported that PD-L1, regulated by lactic acid, plays an important role in promoting immunosuppression in the tumour microenvironment [42]. In addition, PD-L1 expression on LECs inhibits tumour-specific CD8^+^ T-cell responses [43]. Given that lactic acid is the final product of anaerobic glycolysis and HK2 is the key regulator of glycolysis, we examined whether lactic acid production and PD-L1 expression in LECs can be regulated by FGFR and VEGFR inhibition. First, we found that both FGF2 and VEGFC induced lactic acid production in LECs (Fig. 6A). As expected, the lactate concentration was significantly decreased after combined treatment of LECs with infigratinib and SAR131675 compared to single treatment (Fig. 6B). Flow cytometric analysis showed that membrane PD-L1 expression was remarkably downregulated in the combination therapy group (Fig. 6C). Compared with that in the vector control group, membrane PD-L1 expression on LECs transfected with HK2 shRNA was dramatically suppressed (Fig. 6D). Next, whether the combination therapy suppresses the tumour progression in iCCA was investigated in vivo. T cells were injected into established immunodeficient B-NDG mice, bearing iCCA cell-derived tumours. Trial groups were treated plus infigratinib, SAR131675 or the combination. The results showed that both the volumes and weights of tumours were lower in mice that were treated with T cells plus infigratinib or SAR131675 alone or in combination, than in the control mice without any treatments (Fig. 6E). Moreover, immunohistochemical and immunofluorescence assays confirmed higher levels of CD8^+^ T-cell infiltration and lower PD-L1 expression in LECs in the mice treated with T cells plus the combined therapy than in the mice treated with or without T cells plus either infigratinib or SAR131675 (Fig. 6F). The NF-Κb/Cox-2 pathway is reported to be associated with the regulation of PD-L1 expression by lactic acid [11]. The western blot results showed that NF-Κb and Cox-2 expression in LECs was significantly decreased after the combined therapy (Sup Fig. 6A). Consistent with this finding, PD-L1 expression in LECs was decreased when infigratinib and SAR131675 were combined (Sup Fig. 6A). Together, these findings suggest that dual FGFR and VEGFR inhibition improves antitumour immunity in iCCA by downregulating PD-L1 expression in LECs.

**Fig. 7:**
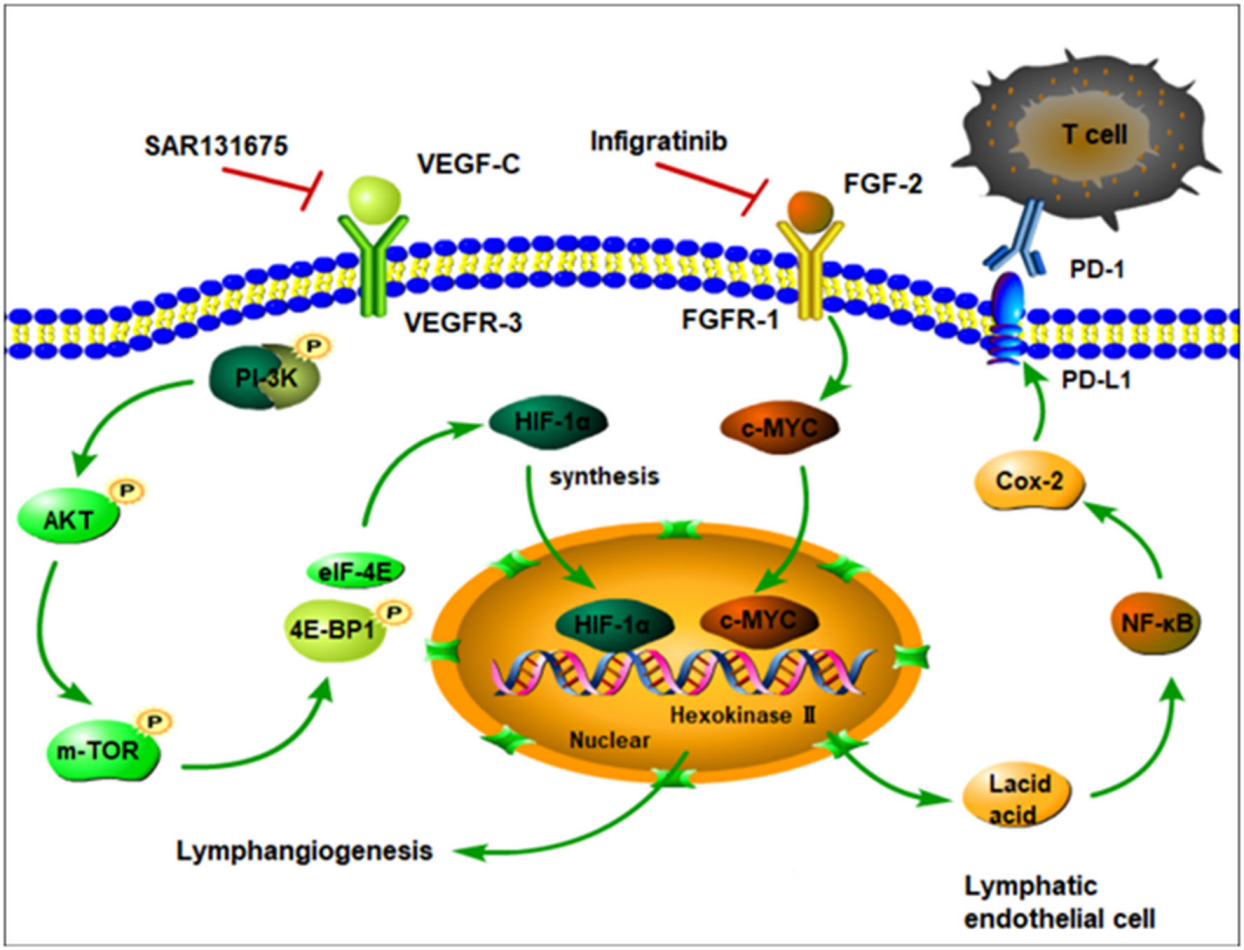
Graphical abstract of FGF and VEGF function in lymphatic endothelial cell. FGF2-FGFR1 pathway and VEGFC-VEGFR3 pathway regulate lymphangiogenesis through promote MYC-dependent and HIF-1α-mediated HK2 expression, respectively. Mechanistically, the VEGFC signalling system regulates HK2 expression by phosphorylating PI3K/Akt/mTOR pathway proteins that regulate HIF-1α expression. Glycolysis activity further influences lactate production, which regulates PD-L1 expression through NF-Κb/Cox-2 pathway. Combination infigratinib and SAR131675 almost completely inhibit lymphangiogenesis and PD-L1 expression by eliminating HK2 expression and reducing lactate production

## Discussion

Lymph node metastasis not only impairs the efficacy of curative treatments but also associated with tumour recurrence and worse outcomes in iCCA patients [2, 44]. Tumour-induced lymphangiogenesis plays an important role in the promotion of cancer metastasis to lymph nodes [45]. Therefore, finding a feasible and effective treatment method to inhibit lymphangiogenesis is an important task for improving the outcomes iCCA patients. In this study, we provide several unique mechanistic insights into lymphangiogenesis by controlling HK2 expression regulated by FGF and VEGF. FGF2 promotes the proliferation, migration and tube formation of LECs by modulating c-MYC-mediated HK2 expression. Here, we originally investigated the inhibitory effects of infigratinib on lymphangiogenesis. Infigratinib, which has shown strong inhibitory effects and promising application prospects in urothelial carcinoma with FGFR3 gene mutation and locally advanced or metastatic gastric cancer with FGFR2 gene amplification, was approved by the FDA for advanced cholangiocarcinoma with FGFR2 fusion and rearrangement in May 2021 and is currently in clinical trials for first-line treatment of cholangiocarcinoma [20, 46–48]. However, the inhibitory effects of infigratinib in LECs are still poorly understood. Our results showed that infigratinib can inhibit LEC growth, migration and tube formation in vitro and lymphangiogenesis in vivo by suppressing HK2 expression. However, treating LECs with infigratinib at the maximum dose or even knocking out the FGFR1 gene in LECs cannot completely inhibit LECs growth and block HK2 expression. Therefore, whether there are any other signalling pathways that influence lymphangiogenesis by regulating HK2 expression is an important question raised by these findings.

Second, we revealed that VEGFC can promote HK2 expression in LECs. This effect may not be reproduced by other lymphangiogenic signalling molecules [16]. VEGFC increased LEC growth, migration and tube formation in vitro and lymphangiogenesis in vivo. Our studies showed that HIF-1α plays a key role in the VEGFR3 signalling system-mediated regulation of HK2 expression and provide evidence that HIF-1α directly binds to the promoter region of HK2. Furthermore, we found that VEGFC can promote HIF-1α expression at the translational level by phosphorylation of PI3K/AKT/mTOR pathway proteins. Specifically, phosphorylated mTOR further phosphorylates 4eBP1 and phosphorylated 4eBP1 (p-4eBP1) can dissociated from eukaryotic translation initiation factor 4E (eIF4E), which relieves the inhibition of protein synthesis. Moreover, P70 S6 kinase can also be phosphorylated by mTOR and then active 40S ribosomal proteins. In the end, activated eIF4E and 40S ribosomal protein cooperatively initiate HIF-1α mRNA translation [40, 49]. To our knowledge, this is the first indication that VEGFC has crucial roles in mediating HK2 expression in LECs.

Based on the above results, we hypothesized that blocking FGFR1 and VEGFR3 may thoroughly inhibit lymphangiogenesis in iCCA. Indeed, we initially observed that treatment of LECs with the combination of infigratinib and SAR131675 at lower therapeutic doses resulted in dramatically decreased LEC growth, migration and tube formation compared with the single agents. HK2 expression in the combination treatment group was also almost completely blocked. Moreover, treatment with infigratinib combined with SAR131675 almost completely inhibited tumour-related lymphangiogenesis in vivo in the iCCA mouse model. Since infigratinib has been approved for the treatment of cholangiocarcinoma patients with FGFR2 gene mutations, cholangiocarcinoma patients who receives this combination therapy may benefit from the inhibition of tumour cell growth and lymphangiogenesis, although more experiments and clinical trials are required to verify our hypothesis.

In addition, we used an intrahepatic cholangiocarcinoma tissue microarray to investigate the correlations between FGFR1, VEGFR3 and HK2 expression and tumour-associated lymphangiogenesis and lymph node metastasis in patients with iCCA. We found that higher expression of HK2, FGFR1 and VEGFR3 in lymphatic vessels was significantly associated with more robust lymphangiogenesis and indicated a worse prognosis. In the lymphatic vessels of iCCA patients, FGFR1 expression and VEGFR3 expression are significantly correlated with HK2 expression and lymphangiogenesis, respectively. Tumour-associated lymphangiogenesis has been reported to be critically involved in lymph node metastasis [6]. Early lymph node metastasis not only makes patients with iCCA lose the opportunity for radical resection, but also tends to be accompanied by a shorter time to postoperative recurrence and a shorter survival time [44]. Therefore, our studies indicated that high expression of FGFR1 and VEGFR3 in lymphatic vessels of iCCA patients may be considered poor prognostic factors. For patients at high risk, early prophylactic treatment may be beneficial to survival.

Lymphatic vessels are challenged by relatively hypoxic conditions [50]. Thus, anaerobic glycolysis is the crucial bioenergetic pathway for LECs [12]. Lactic acid produced via anaerobic glycolysis often promotes PD-L1 expression, which is related to immune escape [51]. Therefore, PD-L1 expression regulated by lactic acid is an important target for converting the tumour environment from an immunosuppressive state to an immunostimulatory state [52]. In our study, the expression of HK2, a key regulator of glycolysis, was dramatically decreased in LECs treated with the combination therapy. Impairing glycolysis further inhibits lactate production, which regulates PD-L1 expression through the NF-ΚB/Cox-2 pathway. The combination of infigratinib and SAR131675 treatment noticeably reduced PD-L1 expression in LECs, an effect that can greatly improve antitumour immune activity.

In general, the FGF2-FGFR1 and VEGFC-VEGFR3 signalling pathways enhanced lymphangiogenesis through MYC-dependent and HIF-1α-mediated HK2 expression, respectively, and the increased glycolytic activity further promoted PD-L1 expression through the NF-Κb/Cox2 pathway. (Fig. 7). The combination of infigratinib with SAR131675 inhibited lymphangiogenesis in vitro and in vivo, and consequently attenuated PD-L1 expression in LECs. These findings may not only identify reliable approaches to block tumour-associated lymphangiogenesis but also improve antitumour immunocompetence in patients with iCCA. In summary, our research may provide a new therapeutic strategy for iCCA.

## Supplementary Information

Below is the link to the electronic supplementary material.

Supplementary file1 (TIF 73 kb)

Supplementary file2 (TIF 56 kb)

Supplementary file3 (TIF 58 kb)

Supplementary file4 (TIF 50 kb)

Supplementary file5 (TIF 312 kb)

Supplementary file6 (TIF 53 kb)

Supplementary file7 (DOCX 35 kb)

## Abbreviations

iCCA: Intrahepatic cholangiocarcinoma
LEC: Lymphatic endothelial cell
HK2: Hexokinase 2
FGFR: Fibroblast growth factor receptor
VEGFR: Vascular endothelial growth factor receptor
