# Supplementary figures and images for: Dual FGFR and VEGFR inhibition synergistically restrain hexokinase 2-dependent lymphangiogenesis and immune escape in intrahepatic cholangiocarcinoma
Source: J Gastroenterol. 2023 Jul 11;58(9):908–24. doi: 10.1007/s00535-023-02012-8 (PMC10423168; doi:10.1007/s00535-023-02012-8)

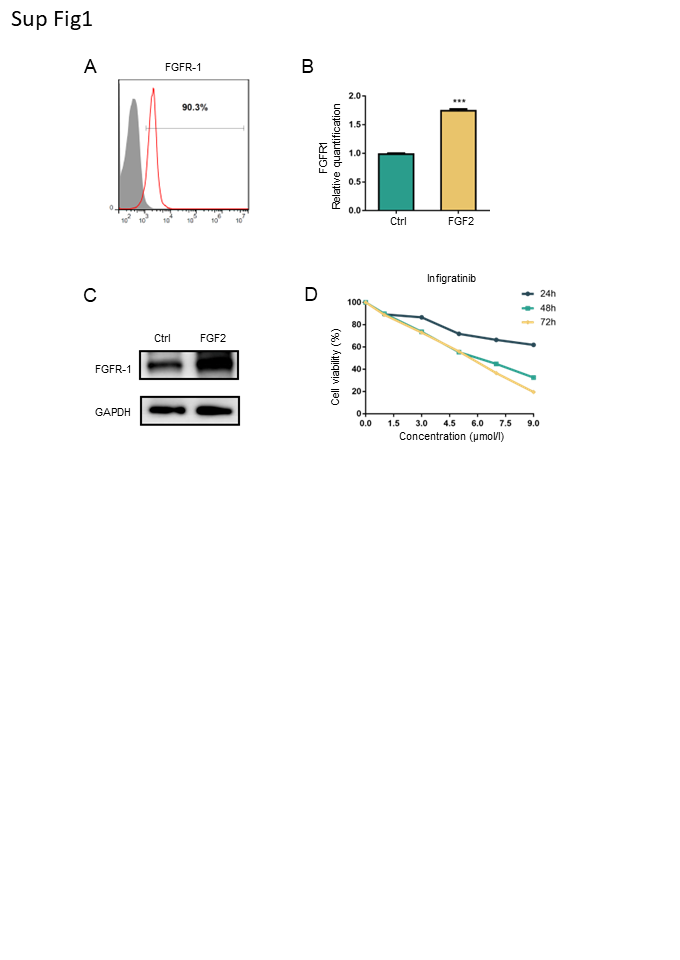

Supplement: Supplementary file 1 — Supplementary file1 (TIF 73 kb) [file 535_2023_2012_MOESM1_ESM.tif]

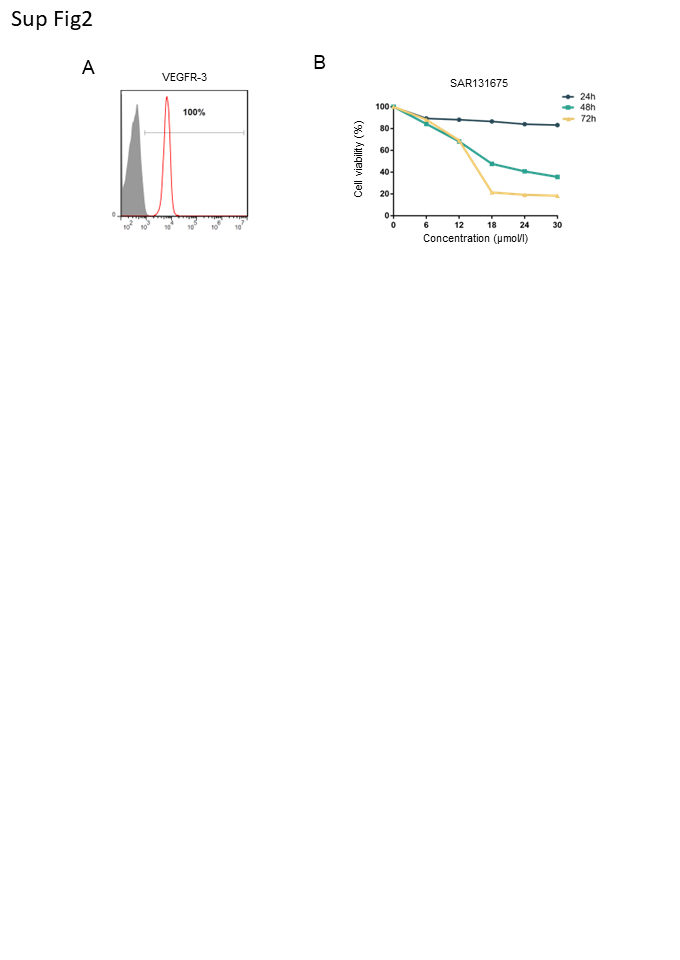

Supplement: Supplementary file 2 — Supplementary file2 (TIF 56 kb) [file 535_2023_2012_MOESM2_ESM.tif]

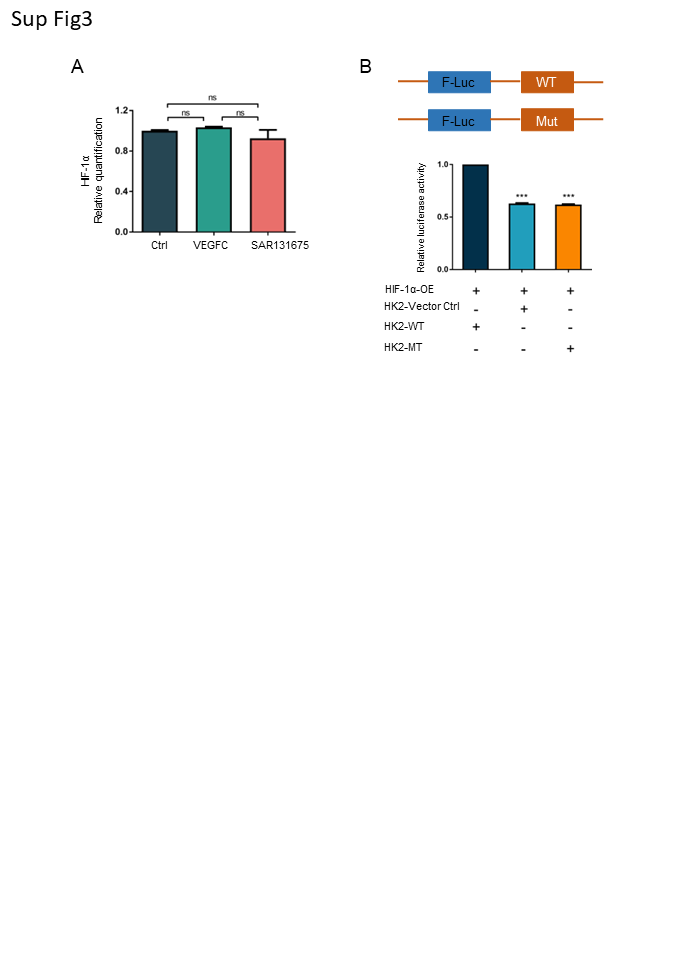

Supplement: Supplementary file 3 — Supplementary file3 (TIF 58 kb) [file 535_2023_2012_MOESM3_ESM.tif]

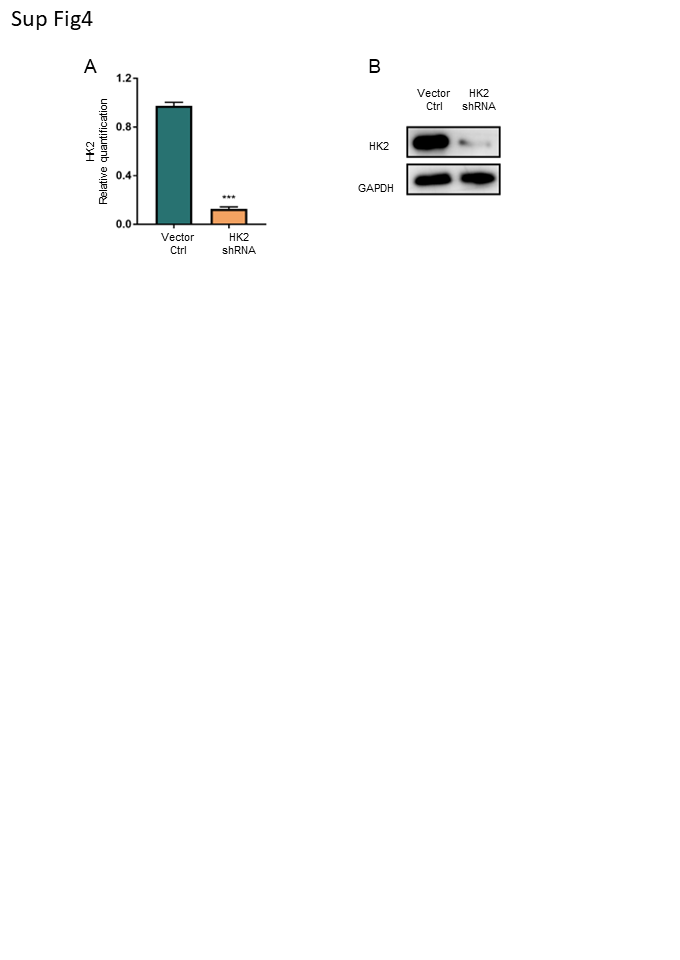

Supplement: Supplementary file 4 — Supplementary file4 (TIF 50 kb) [file 535_2023_2012_MOESM4_ESM.tif]

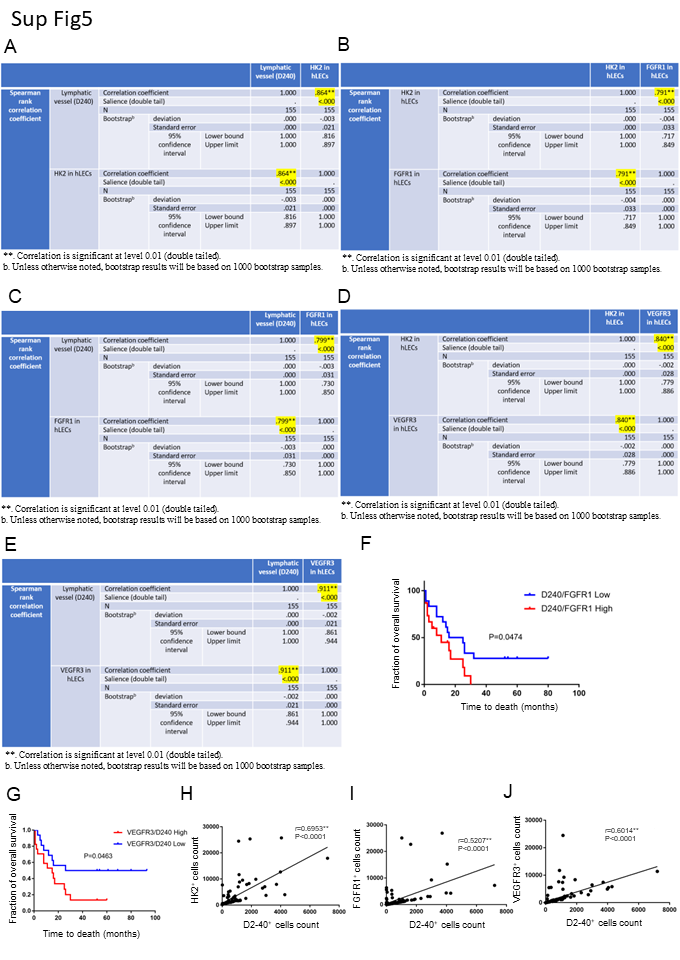

Supplement: Supplementary file 5 — Supplementary file5 (TIF 312 kb) [file 535_2023_2012_MOESM5_ESM.tif]

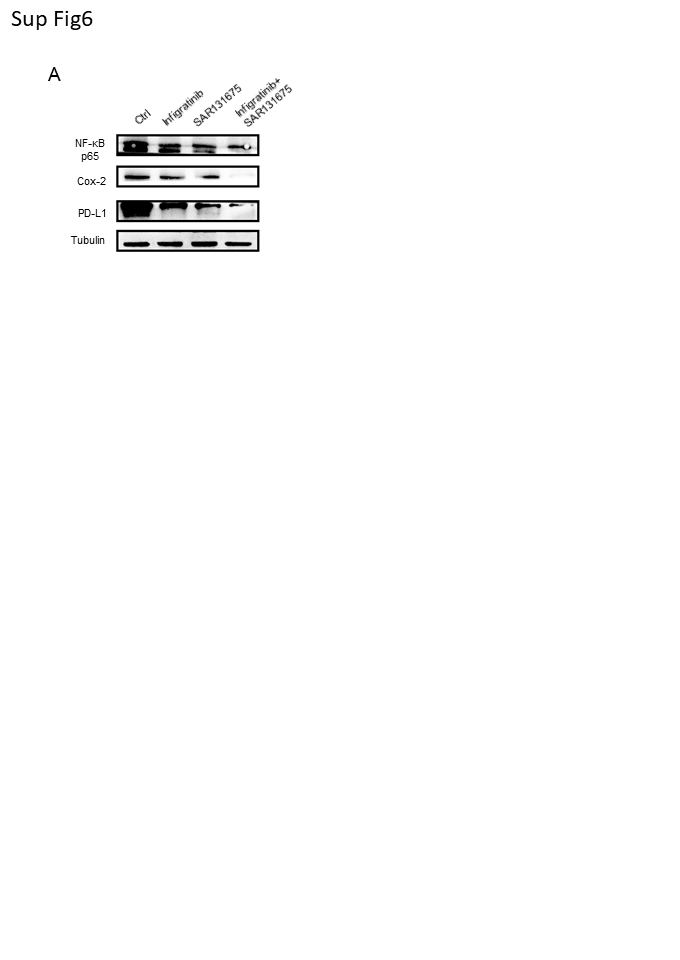

Supplement: Supplementary file 6 — Supplementary file6 (TIF 53 kb) [file 535_2023_2012_MOESM6_ESM.tif]
