# Supplementary material for: Dual FGFR and VEGFR inhibition synergistically restrain hexokinase 2-dependent lymphangiogenesis and immune escape in intrahepatic cholangiocarcinoma
Source: J Gastroenterol. 2023 Jul 11;58(9):908–24. doi: 10.1007/s00535-023-02012-8 (PMC10423168; doi:10.1007/s00535-023-02012-8)
Supplement: Supplementary file 7 — Supplementary file7 (DOCX 35 kb) [file 535_2023_2012_MOESM7_ESM.docx]

Sup table 1

A total of 155 cases of iCCA cancer tissue were included in the analysis.

Detection index：D240 antibody, FGFR1 antibody, VEGFR3 antibody, HK2 antibody

Antibodies detection results were as follows:

| Variables | Number | | | total |
| --- | --- | --- | --- | --- |
|  | - | low | high |  |
| D2-40^+^ | 20 | 40 | 95 | 155 |
| D2-40^+^FGFR1^+^ | 66 | 56 | 33 | 155 |
| D2-40^+^VEGFR3^+^ | 34 | 60 | 61 | 155 |
| D2-40^+^HK2^+^ | 52 | 34 | 69 | 155 |

D2-40^+^： - was represented number=0 (-)；low was represented 0<number≤41 (low)；high was represented number>41 (high).

D2-40^+^FGFR1^+^： - was represented number=0 (-)；low was represented 0<number≤23 (low)； high was represented number>23 (high).

D2-40^+^VEGFR3^+^： - was represented number=0 (-)；low was represented 0<number≤58 (low)； high was represented number>58 (high).

D2-40^+^HK2^+^： - was represented number=0 (-)；low was represented 0<number≤10 (low)； high was represented number>10 (high).

Sup table 2

Correlation between D2-40^+^FGFR1^+^ and clinicopathological characteristics

|  | variables | D2-40+FGFR1+ | | | total | χ^2^ | p value |
| --- | --- | --- | --- | --- | --- | --- | --- |
|  |  | - | low | high |  |  |  |
| Age (year) |  |  |  |  |  | 4.350 | 0.114 |
|  | ≤60 | 34 | 26 | 10 | 70 |  |  |
|  | >60 | 31 | 29 | 23 | 83 |  |  |
| Sex |  |  |  |  |  | 2.498 | 0.287 |
|  | Female | 27 | 17 | 15 | 59 |  |  |
|  | male | 38 | 39 | 18 | 95 |  |  |
| Grade |  |  |  |  |  | 8.132 | 0.017 |
|  | I/II | 33 | 28 | 25 | 86 |  |  |
|  | III | 33 | 28 | 7 | 68 |  |  |
| T stage |  |  |  |  |  | 0.937 | 0.626 |
|  | T1/T2 | 19 | 6 | 6 | 31 |  |  |
|  | T3/T4 | 3 | 0 | 1 | 4 |  |  |
| N stage |  |  |  |  |  | 12.795 | 0.002 |
|  | N0 | 20 | 15 | 2 | 37 |  |  |
|  | N1/N2 | 4 | 4 | 7 | 15 |  |  |
| M stage |  |  |  |  |  | 0.261 | 0.878 |
|  | M0 | 63 | 53 | 32 | 148 |  |  |
|  | M1 | 3 | 3 | 1 | 7 |  |  |
| TNM stage |  |  |  |  |  | 1.766 | 0.414 |
|  | Ι/II | 14 | 3 | 5 | 22 |  |  |
|  | III/IV | 12 | 7 | 7 | 26 |  |  |
| [Tumor](D:/Program%20Files%20(x86)/Youdao/Dict/8.9.6.0/resultui/html/index.html#/javascript:;) [size](D:/Program%20Files%20(x86)/Youdao/Dict/8.9.6.0/resultui/html/index.html#/javascript:;) |  |  |  |  |  | 4.082 | 0.13 |
|  | ≤5cm | 27 | 15 | 17 | 59 |  |  |
|  | >5cm | 36 | 32 | 14 | 82 |  |  |
| Number of lymph nodes |  |  |  |  |  | 4.414 | 0.11 |
|  | ≤1 | 4 | 4 | 7 | 15 |  |  |
|  | >1 | 16 | 11 | 6 | 33 |  |  |
| Positive lymph node |  |  |  |  |  | 2.131 | 0.345 |
|  | ≤0 | 20 | 16 | 8 | 44 |  |  |
|  | >0 | 12 | 5 | 7 | 24 |  |  |

P-values were calculated using Pearson chi-square test. P-values﹤0.05 was regarded as statistically significant.

- was represented as number=0 (-)；low was represented as 0<number≤23 (low)；high was represented as number>23 (high).

Sup table 3

Correlation between D2-40^+^VEGFR3^+^ and clinicopathological characteristics

|  | variables | D2-40^+^VEGFR3^+^ | | | total | χ^2^ | p value |
| --- | --- | --- | --- | --- | --- | --- | --- |
|  |  | - | low | high |  |  |  |
| Age (year) |  |  |  |  |  | 0.719 | 0.698 |
|  | ≤60 | 13 | 29 | 28 | 70 |  |  |
|  | >60 | 20 | 31 | 32 | 83 |  |  |
| Sex |  |  |  |  |  | 1.538 | 0.463 |
|  | Female | 11 | 21 | 27 | 59 |  |  |
|  | male | 22 | 39 | 34 | 95 |  |  |
| Grade |  |  |  |  |  | 16.257 | <0.0001 |
|  | I/II | 10 | 32 | 44 | 86 |  |  |
|  | III | 24 | 27 | 17 | 68 |  |  |
| T stage |  |  |  |  |  | 1.612 | 0.447 |
|  | T1/T2 | 7 | 14 | 10 | 31 |  |  |
|  | T3/T4 | 0 | 3 | 1 | 4 |  |  |
| N stage |  |  |  |  |  | 9.645 | 0.008 |
|  | N0 | 8 | 21 | 8 | 37 |  |  |
|  | N1/N2 | 1 | 4 | 10 | 15 |  |  |
| M stage |  |  |  |  |  | 2.666 | 0.264 |
|  | M0 | 31 | 57 | 60 | 148 |  |  |
|  | M1 | 3 | 3 | 1 | 7 |  |  |
| TNM stage |  |  |  |  |  | 1.223 | 0.543 |
|  | Ι/II | 4 | 11 | 7 | 22 |  |  |
|  | III/IV | 7 | 9 | 10 | 26 |  |  |
| [Tumor](D:/Program%20Files%20(x86)/Youdao/Dict/8.9.6.0/resultui/html/index.html#/javascript:;) [size](D:/Program%20Files%20(x86)/Youdao/Dict/8.9.6.0/resultui/html/index.html#/javascript:;) |  |  |  |  |  | 0.198 | 0.906 |
|  | ≤5cm | 12 | 23 | 24 | 59 |  |  |
|  | >5cm | 19 | 32 | 31 | 82 |  |  |
| Number of lymph nodes |  |  |  |  |  | 13.535 | 0.001 |
|  | ≤1 | 3 | 1 | 11 | 15 |  |  |
|  | >1 | 3 | 21 | 9 | 33 |  |  |
| Positive lymph node |  |  |  |  |  | 1.755 | 0.416 |
|  | ≤0 | 8 | 22 | 14 | 44 |  |  |
|  | >0 | 6 | 8 | 10 | 24 |  |  |

P-values were calculated using Pearson chi-square test. P-values﹤0.05 was regarded as statistically significant.

- was represented as number=0 (-)；low was represented as 0<number≤58 (low)；high was represented as number>58 (high).

Sup table 4

Correlation between D2-40^+^HK2^+^ and clinicopathological characteristics

|  | variables | D2-40^+^HK2^+^ | | | total | χ^2^ | p value |
| --- | --- | --- | --- | --- | --- | --- | --- |
|  |  | - | low | high |  |  |  |
| Age (year) |  |  |  |  |  | 0.601 | 0.74 |
|  | ≤60 | 22 | 17 | 31 | 70 |  |  |
|  | >60 | 29 | 16 | 38 | 83 |  |  |
| Sex |  |  |  |  |  | 1.424 | 0.491 |
|  | Female | 18 | 16 | 25 | 59 |  |  |
|  | male | 33 | 18 | 44 | 95 |  |  |
| Grade |  |  |  |  |  | 4.262 | 0.119 |
|  | I/II | 24 | 18 | 44 | 86 |  |  |
|  | III | 28 | 16 | 24 | 68 |  |  |
| T stage |  |  |  |  |  | 1.889 | 0.389 |
|  | T1/T2 | 12 | 6 | 13 | 31 |  |  |
|  | T3/T4 | 1 | 2 | 1 | 4 |  |  |
| N stage |  |  |  |  |  | 9.732 | 0.008 |
|  | N0 | 15 | 11 | 11 | 37 |  |  |
|  | N1/N2 | 0 | 5 | 10 | 15 |  |  |
| M stage |  |  |  |  |  | 0.755 | 0.686 |
|  | M0 | 49 | 32 | 67 | 148 |  |  |
|  | M1 | 3 | 2 | 2 | 7 |  |  |
| TNM stage |  |  |  |  |  | 0.233 | 0.89 |
|  | Ι/II | 9 | 5 | 8 | 22 |  |  |
|  | III/IV | 9 | 6 | 11 | 26 |  |  |
| [Tumor](D:/Program%20Files%20(x86)/Youdao/Dict/8.9.6.0/resultui/html/index.html#/javascript:;) [size](D:/Program%20Files%20(x86)/Youdao/Dict/8.9.6.0/resultui/html/index.html#/javascript:;) |  |  |  |  |  | 3.211 | 0.201 |
|  | ≤5cm | 17 | 10 | 32 | 59 |  |  |
|  | >5cm | 32 | 18 | 32 | 82 |  |  |
| Number of lymph nodes |  |  |  |  |  | 3.361 | 0.186 |
|  | ≤1 | 4 | 1 | 10 | 15 |  |  |
|  | >1 | 10 | 9 | 14 | 33 |  |  |
| Positive lymph node |  |  |  |  |  | 0.206 | 0.902 |
|  | ≤0 | 15 | 11 | 18 | 44 |  |  |
|  | >0 | 8 | 5 | 11 | 24 |  |  |

P-values were calculated using Pearson chi-square test. P-values﹤0.05 was regarded as statistically significant.

- was represented as number=0 (-)；low was represented as 0<number<10 (low)；high was represented as number≥10 (high).
